# Tubular organotypic culture model of human kidney

**DOI:** 10.1371/journal.pone.0206447

**Published:** 2018-10-31

**Authors:** Dae-young Jun, Sook Young Kim, Joon Chae Na, Hyung Ho Lee, Jeehoon Kim, Young Eun Yoon, Sung Joon Hong, Woong Kyu Han

**Affiliations:** 1 Department of Urology, Urological Science Institute, Yonsei University College of Medicine, Seoul, Republic of Korea; 2 Department of Urology, National Health Insurance Service Ilsan Hospital, Gyeonggi-do, Republic of Korea; 3 Department of Urology, Hanyang University College of Medicine, Seoul, Republic of Korea; 4 Brain Korea 21 PLUS Project for Medical Science, Yonsei University College of Medicine, Seoul, Republic of Korea; University of Kansas Medical Center, UNITED STATES

## Abstract

Cell-culture methods that simplify the inherent complexities of the kidney have not sufficiently reproduced its true characteristics. Although reports indicate that organoid methodology surpasses traditional cell culture in terms of reproducing the nature of organs, the study of human kidney organoids have been confined to pluripotent stem cells. Furthermore, it has not yet progressed beyond the developmental state of embryonic kidney even after complicate additional differentiation processes. We here describe the kidney organotypic culture method that uses adult whole kidney tissues but mainly differentiates into tubular cells. This model was validated based on the retention of key kidney organotypic-specific features: 1) expression of Tamm-Horsfall protein; 2) dome-like organoid configurations, implying directed transport of solutes and water influx; and 3) organoid expression of neutrophil gelatinase-associated lipocalin (NGAL) and kidney injury molecule-1 (KIM-1) in response to nephrotoxic injury (i.e., gentamicin and cisplatin exposure). This 3D-structured organoid prototype of the human renal tubule may have applications in developing patient-specific treatments for kidney diseases.

## Introduction

The global prevalence of chronic kidney disease is 13.4%, which represents a major cost burden to healthcare systems worldwide [1]. At present, patient management remains limited to reducing inflammation, optimizing cardiovascular risk, and providing supportive care. A better understanding of physiologic and pathophysiologic mechanisms involved in renal damage and repair will likely promote therapeutic advancements [2].

The human kidney harbors a number of cell types, and its architecture is complex, posing a challenge for *in vivo* studies. Although two-dimensional (2D) cell-culture models have value in the study of renal pathophysiology, the superiority of three-dimensional (3D) culture models that simulate the *in vivo* environment is readily acknowledged [2]. Therefore, an effective organoid model that mimics the human kidney is a welcomed contribution. Organoids reflect key structural and functional properties of bodily organs, providing a potential means of replicating human organ pathologies “in a dish” [3].

Such 3D organoids have been derived from tissue-specific progenitors, induced pluripotent stem cells (iPSCs), or embryonic stem cells (ESCs) to mimic a host or human organs, including brain [4], retina [5], stomach [6], small intestine [6], lung [7], thyroid [8], liver [9], pancreas [10], and kidney [11]; and their features are distinctive. Organoid models incorporate multiple organ-specific cell types, recapitulating organ development and function. They also self-organize through cell sorting and spatially restricted lineage commitment in manners similar to *in vivo* events [12]. The therapeutic ramifications of organoid technology extend to infectious disease [13], hereditary disorders [4], drug-related toxicity [14], tumorogenesis [15], and organ transplantation [16]. With personalized medicine as the goal, identifying and implementing successful patient treatments seem feasible [3].

Although the vast potential of organoids is abundantly clear, one must bear in mind the current constraints. They lack innervation, vascularization, and immune cells, so diseases states under study are incompletely reproduced [3]. Furthermore, the use of human ESCs raises ethical concerns, and the clinical utility of iPSC-derived organoids is undermined by tumorigenic risk [17]. Resolution of these issues no doubt would heighten the use of organoids in research fields and clinics [17, 18]. Indeed, such problems could be circumvented by using normal human kidney cells for organoid experimentation, which to our knowledge has yet to be pursued.

Stem cell organoids consume substantial amounts of time and funding due to the many growth factors needed and various differentiation stages that must occur. Even so, these studies have yielded nothing more than progenitor kidney, and experimental models of this nature are far removed from clinical practice. The present investigation was undertaken to generate an efficient organoid model from adult human kidney tissue. Ultimately, expression of kidney-specific proteins and replication of 3D tubular structure by organoid constituents were used for validation.

## Materials and methods

### Human tissues and primary tubule cells

Normal renal tissues were collected from patients who provided informed consent as stipulated by the Yonsei University Health System, Severance Hospital, Institutional Review Board, and the study protocol was approved by the same institutional review board (approval number 4-2015-0104). Primary normal human RPTECs were purchased from the American Type Culture Collection (ATCC, PCS-400-010) and were maintained (37°C, 5% CO2) in renal epithelial cell growth media (ATCC, PCS-400-040) containing 0.5% fetal bovine serum (FBS), 10 nM triiodothyronine, 10 ng/ml recombinant human EGF, 100 ng/ml hydrocortisone, 5 μg/ml recombinant human insulin, 1 μM epinephrine, 5 μg/ml transferrin, and 2.4 mM L-alanyl-L-glutamine.

### Organotypic culture

Using a blade, dissected human kidney samples were minced into 1 × 1-mm pieces, then incubated (37°C, 2 h) in 5 ml of Dulbecco’s Modified Eagle Medium/Nutrient Mixture F-12 (DMEM-F12; Gibco [Thermo Fisher], Grand Island, NY, USA), supplemented with 1% FBS, 3 mg/ml collagenase type II (Sigma-Aldrich, St Louis, MO, USA), and 1× antibiotic/antimycotic solution (Sigma-Aldrich) to allow for tissue dissociation/degradation. Thereafter, the product was triturated vigorously by pipetting (1 min) and filtering through a 70-μm cell strainer (Corning Corp, Corning, NY, USA). Cell pellets from subsequent centrifugation (~200 g, 2 min) were gently washed twice in phosphate-buffered saline (PBS). We used two methods to culture kidney organoids: the membrane matrix (Matrigel; Corning Inc, Corning, NY, USA) 3D-embedded method and the 3D-on-top assay, a more cost-effective alternative [19]. Briefly, 1–2 ml of Matrigel (Corning) was added to each cell pellet (approximately 1x10^3^ cells/μl of matrigel) within a 6-well culture plate (3D embedded) in an organoid substrate of serum-free keratinocyte medium (Gibco) supplemented with 10 ng/ml recombinant human EGF (Gibco), 50 μg/ml bovine pituitary extract (Gibco), 0.01 mg/ml recombinant human insulin, 55 μg/ml human transferrin (substantially iron-free), 5 ng/ml sodium selenite (ITS supplement, Sigma), 500 nM hydrocortisone (Sigma), 100 ng/ml human recombinant Noggin (PeproTech, Rocky Hill, NJ, USA), 10 nM Leucin (Sigma), 5 μM Y-27632 (Enzo Life Sciences, Farmingdale, NY, USA), and 5% FBS. For 3D-on-top culture, cells were plated on Matrigel (freshly molded) within organoid culture medium in a 6-well plate. For serial passage of organoids (every 1–2 weeks), incubation (37° C, 5 min) in a 1:4 dilution of 0.25% trypsin was used, followed by mechanical dissociation to nearly single-cell suspensions (as described by Chua et al) [20].

### Cell proliferation assay (CCK-8 assay)

Human RPTECs and cells from normal renal tissues were loaded in triplicate in 48-well culture plate with matrigel (1x10^4^ cells or 3x10^4^ cells/25μl of each matrigel dom). Cells were incubated for 72hr in renal epithelial cell growth media and organoid media, respectively. The cell proliferation was detected by Cell Counting kit-8 (CCK-8, Dojindo Laboratories, Kumamoto, Japan). 2-(2-methoxy-4-nitrophenyl)-3-(4-nitrophenyl)-5-(2,4-disulfophenyl)-2H-tetrazolium, monosodium salt was added each well and incubated for 1hr. A water soluble formazan product in media was determined using a Beckman Coulter microplate reader at 450nm.

### Immunofluorescent preparations

Expression levels of Tamm-Horsfall protein (THP), pan-keratin (Ker), NGAL, and KIM-1 were measured using immunofluorescence. Briefly, after a 2-week differentiation period, 3D kidney organoids (3D-embedded) in PBS were pipetted very gently to separate them from the Matrigel matrix for whole organoid stain. Whole organoids seeded (37°C, 16 h) onto an 8-chamber cell-culture slide were fixed (10 min) in 4% paraformaldehyde solution and washed in PBS. Cells were permeated (5 min) in 0.5% Triton X-100/PBS, blocked (20 min) with 5% BSA/PBS, and incubated (4°C, 16 h) in diluted primary antibodies: FITC-conjugated anti-THP (Cedarlane Laboratories, Burlington, NC, USA), anti-NGAL (Abcam, Cambridge, UK), anti-KIM-1 (Abcam) and anti-pan-keratin (Cell Signaling Technology, Danvers, MA, USA). Finally, PBS-washed cells were exposed (25° C, 1 h) to secondary antibodies tagged with Alexa Fluor 488 or 594 (1:200; Invitrogen [Thermo Fisher], Waltham, MA, USA). Images were captured using a confocal microscope (LSM 700 META; Carl Zeiss AG, Oberkochen, Germany) and analyzed using proprietary software (LSM Image Browser).

### Acute nephrotoxic injury model

To induce injury, 3D kidney organoids differentiated for 2 weeks (3D-embedded) were incubated (24 h) in 2 mg/ml gentamicin (Sigma) or 5 μM cisplatin (Sigma) and transferred by spatula to 1% agarose in DMEM for immobilization (room temperature, 30 min). Subsequently, cells were fixed in 10% formalin (room temperature, 24 h) for routine histologic processing, paraffin embedding, and sectioning. Upon deparaffinization, morphology was confirmed microscopically by hematoxylin and eosin staining, and expression levels of NGAL and KIM-1, determined by immunofluorescent staining, were used as markers to gauge acute nephrotoxic injury.

### Statistical analysis

Quantitative values were expressed as mean ± standard deviation (SD). Statistical differences were compared via Mann-Whitney U-test, using standard software (IBM SPSS Statistics v23.0; IBM Corp, Armonk, NY, USA) for all computations. A *p*<0.05 was considered statistically significant.

## Results

### Normal human kidney cells form tubulocysts in 3D culture

**Fig 1:**
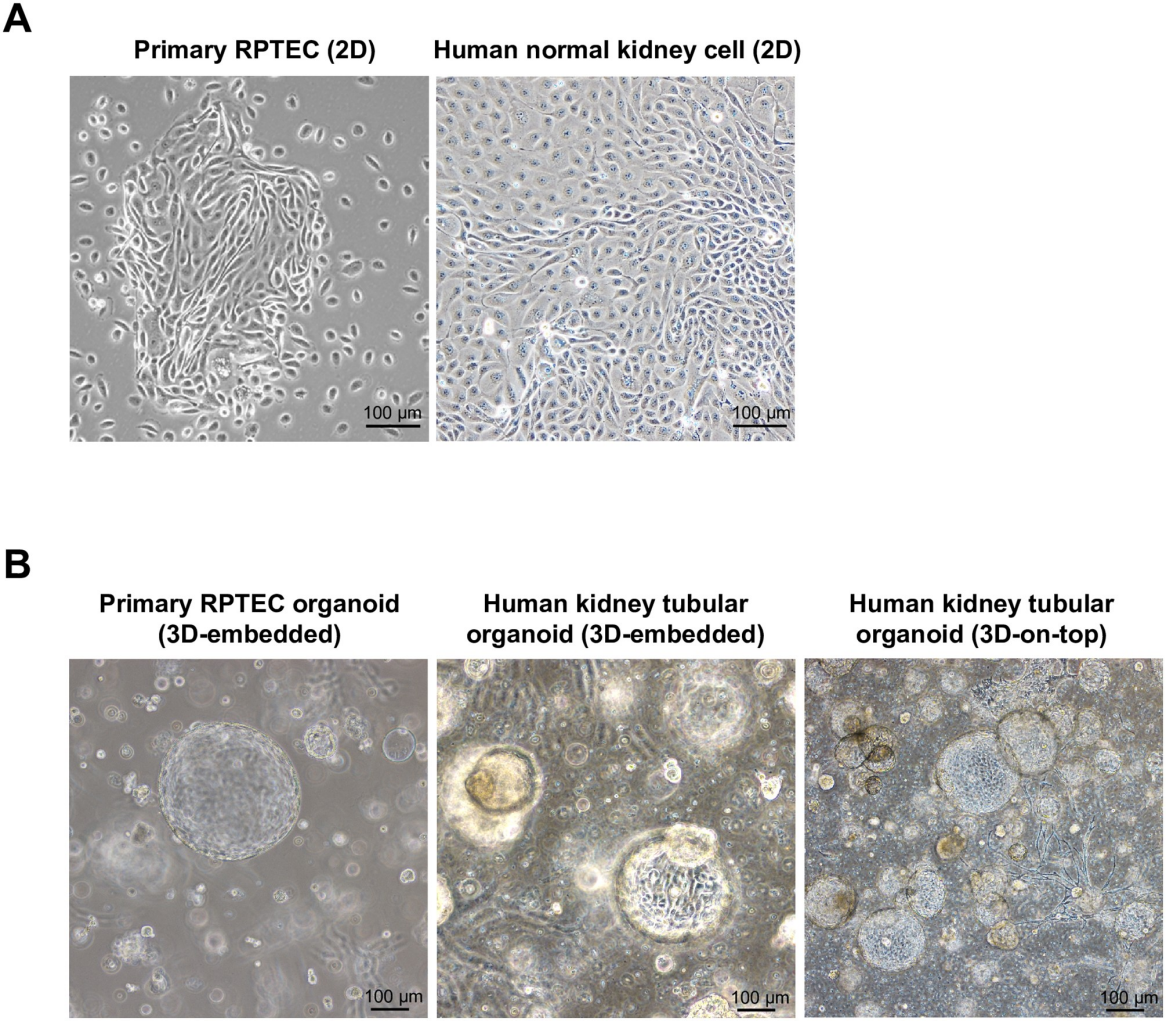
Primary renal proximal tubular epithelial cells (RPTECs) and normal human kidney cells form tubulocysts. (A) Phase-contrast images: primary RPTECs and normal human kidney cells showing similar cellular morphologies in 2D cultures. (B) Phase-contrast images: spherule formation observed in primary RPTECs and normal human kidney cells. The 3D-on-top assay provides a clearer image of human normal kidney cell organoids than the 3D-embedded assay.

Normal human kidney cells exhibited morphologic characteristics similar to primary renal proximal tubular epithelial cells (RPTECs) (ATCC, PCS-400-010), forming tight epithelial layers when grown to confluency in 2D culture (Fig 1A). They also assumed dome-like tubulocystic configurations (called tubulocysts) in 3D culture and 3D-on-top culture (Fig 1B). The 3D-on-top culture was used for cleaner colonies images of human kidney tubular organoids and easy handling with Matrigel than the 3D-embedded assay (Fig 1B) [19].

### Normal human kidney cells generate more tubulocysts than primary renal proximal tubular epithelial cells (RPTECs) (ATCC, PCS-400-010) in organoid culture

**Fig 2:**
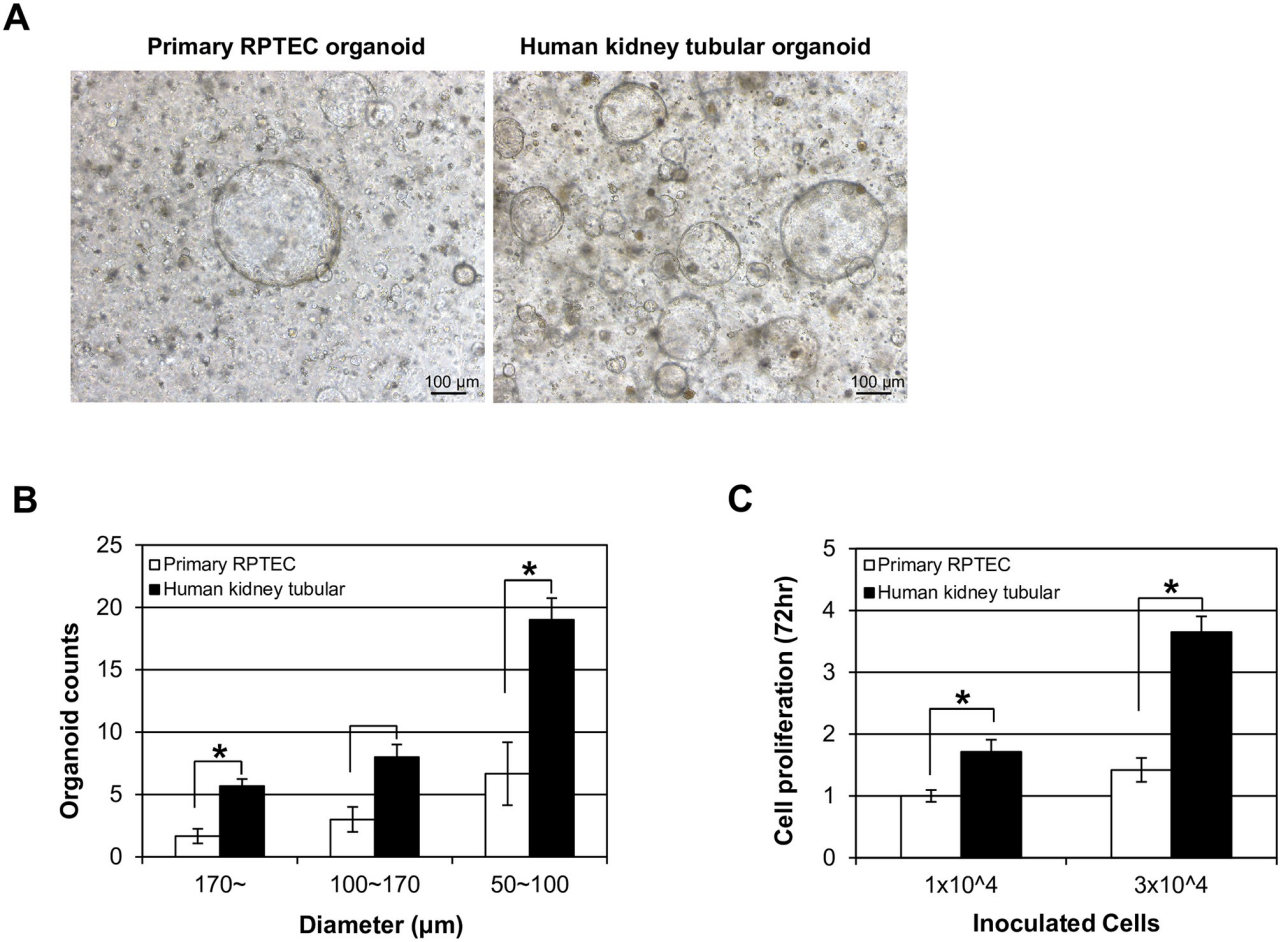
Normal human kidney cells generate more tubulocysts than primary renal proximal tubular epithelial cells (RPTECs). (A) Phase-contrast images: primary RPTECs and normal human kidney cells exhibiting tubulocysts formation. (B) Quantification (counts) of tubulocysts in primary RPTEC and normal human kidney cell organoids, categorized by size. Results expressed as mean (± SD) of three independent experiments (n = 3); *p<0.05 vs control. (C) Proliferation assay of primary RPTECs and normal human kidney cells using CCK8 for 72hr. Each matrigel dome containing 1x10^4^ cells and 3x10^4^ cells/25μl of matrigel were loaded in 48-well culture plates. All experiments were performed in triplicate for each condition and repeated at least twice. *p<0.05 vs control.

Both primary RPTECs and normal human kidney cells formed tubulocysts in organoid cultures (Fig 2A). Tubulocysts counts in human kidney tubular organoids exceeded those of primary RPTEC organoids. When each tubulocyst was categorized by size (diameter), the counts in human kidney tubular organoids greatly surpassing those of primary RPTEC organoids for all categories (≥170 μm, *p* = 0.043; 100–170 μm, *p* = 0.050; 50–100 μm, *p* = 0.046) (Fig 2B). Cell proliferation was also more prominent in human kidney cells compared to RPTEC cells. When the same number of cells (1x10^4^ cells and 3x10^4^ cells/25μl) were loaded, after 72hrs the human kidney cells proliferated 1.4 and 2.6- fold more, respectively, compared to RPTEC cells (Fig 2C).

### Normal human kidney cells express Tamm-Horsfall protein, acquiring morphologic features of renal tubular cells

**Fig 3:**
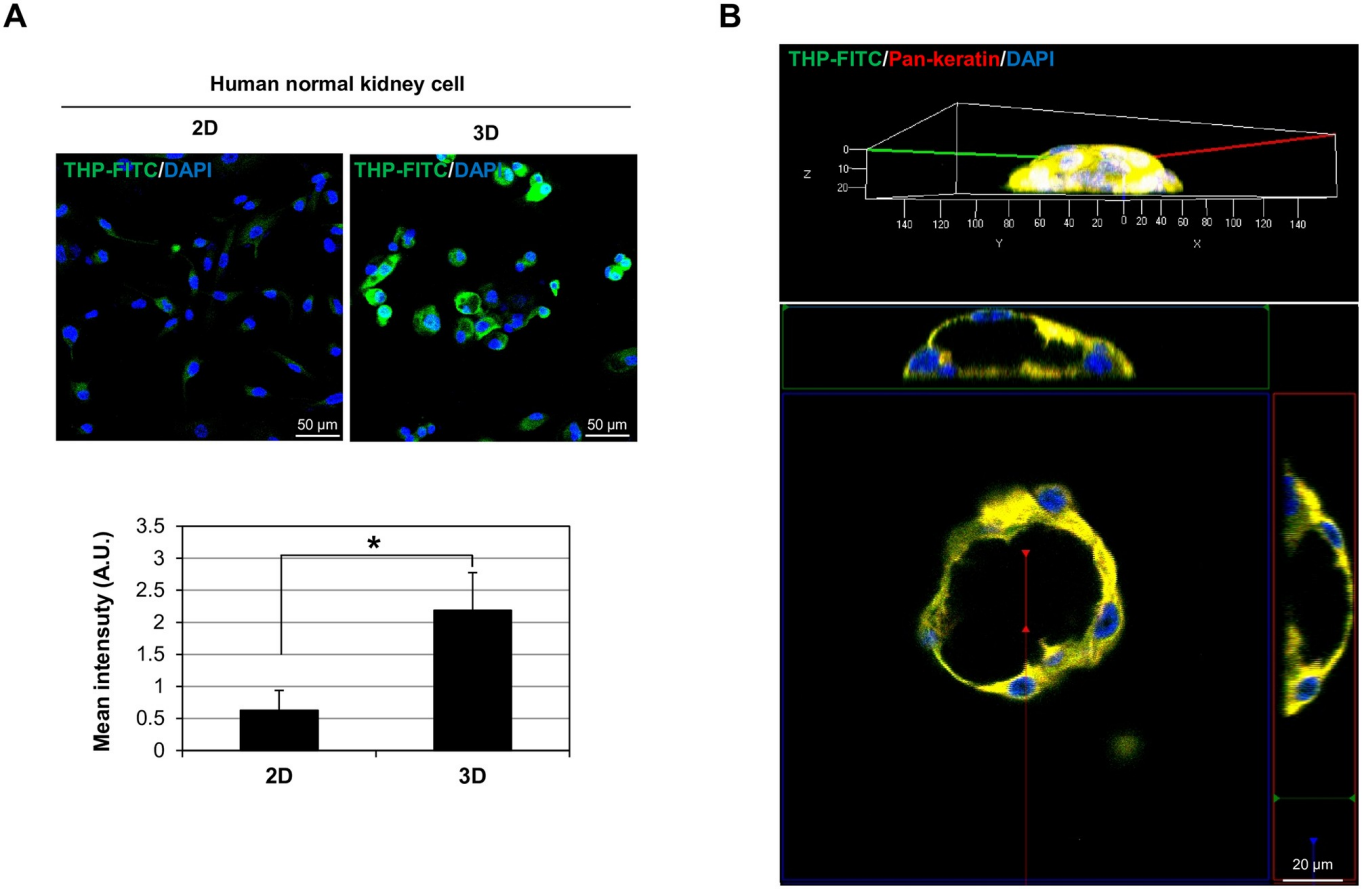
Human kidney tubular organoid expression of kidney-specific molecules and formation of dome-like structures. (A) Confocal images: immunostaining of normal human kidney cells for Tamm-Horsfall protein in 2D and 3D cultures (upper panel); quantification of fluorescence intensity of Tamm-Horsfall protein expression by normal human normal kidney cells in 2D and 3D cultures (lower panel). THP-FITC (green), FITC-conjugated anti-Tamm-Horsfall protein; DAPI (blue), 4′,6-diamidine-2′-phenylindole dihydrochloride. Results expressed as mean (± SD) of five independent experiments (n = 5); *p< 0.05 vs control. (B) 3D reconstruction of confocal z-stacks: immunostaining of mildly dissolved and re-cultured human kidney tubular organoids for Tamm-Horsfall protein. THP-FITC (green), FITC-conjugated anti-Tamm-Horsfall protein; pan-keratin (red), anti-pan-keratin antibody; DAPI (blue), 4′,6-diamidine-2′-phenylindole dihydrochloride.

Normal human kidney cells in 2D or 3D culture expressed Tamm-Horsfall protein, an event typical of renal tubules (Fig 3A) [21, 22]. Tamm-Horsfall protein expression was greater in 3D (vs 2D) cultures of normal human kidney cells (*p* = 0.009) (Fig 3A). These results highlight the relative superiority of 3D culture in simulating one aspect of renal tubules. In addition, the normal human kidney cells formed dome-like structures with central lumens when grown in Matrigel. In these distinctly tubular organoids, expression of Tamm-Horsfall protein was robust (Fig 3B and S1 Video).

### Validation of experimental organoids derived from normal human kidney cells

**Fig 4:**
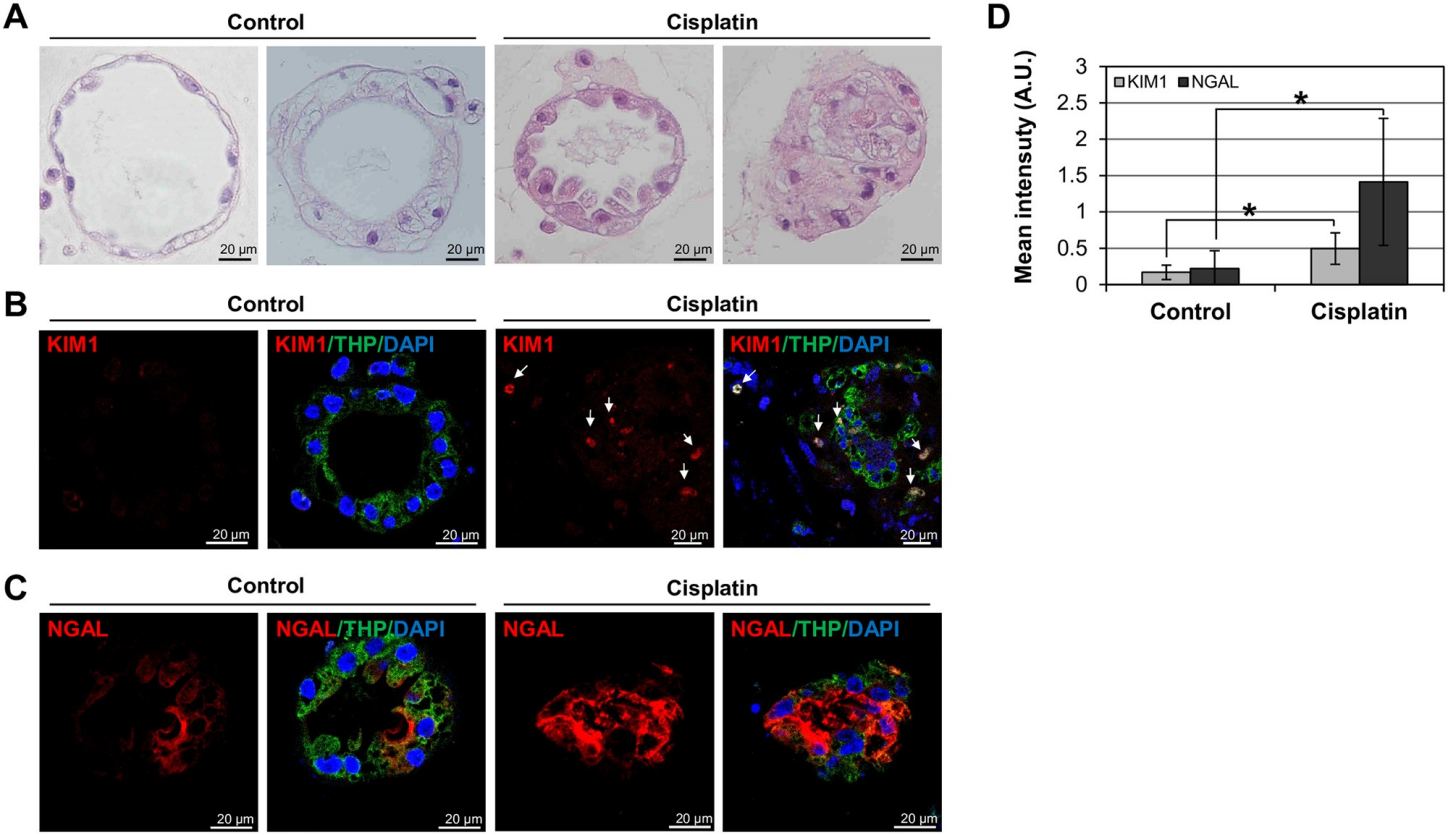
Human kidney tubular organoid response to nephrotoxic drugs. (A) Light microscopy: hematoxylin and eosin staining of human kidney tubular organoid sections, untreated (Control) and cisplatin-treated (Cisplatin). (B, C) Confocal images: immunostaining of kidney injury molecule-1 (KIM-1), neutrophil gelatinase-associated lipocalin (NGAL), and Tamm-Horsfall protein (THP) in untreated (Control) and cisplatin-treated (Cisplatin) human kidney tubular organoid sections. (D) Quantification of fluorescence intensity of KIM-1 and NGAL in untreated (Control) and cisplatin-treated (Cisplatin) human kidney tubular organoids. KIM-1 (red), anti-KIM-1 antibody; NGAL (red), anti-NGAL antibody; THP (green), FITC-conjugated anti-Tamm-Horsfall protein; DAPI (blue), 4′,6-diamidine-2′-phenylindole dihydrochloride. Results expressed as mean (± SD) of five independent experiments (n = 5); *p<0.05 vs control.

Acute renal injury results from damage to renal tubular cells due to frequent exposure to nephrotoxic substances, such as gentamicin and cisplatin. Neutrophil gelatinase-associated lipocalin (NGAL) and kidney injury molecule-1 (KIM-1), specific biomarkers for tubular injury [23], were higher in normal human kidney cells treated with nephrotoxic drugs, compared with controls (Fig 4 and S1 Fig). Slides of human kidney tubular organoids selectively exposed to cisplatin were stained with hematoxylin and eosin (Fig 4A). The integrity of cisplatin-treated tubular organoids was disrupted compared with untreated organoids (Fig 4A). Expression levels of KIM-1 and NGAL were investigated in human kidney tubular organoids, both with and without cisplatin exposure (Fig 4B and 4C). Both markers had significantly higher expression levels (*p* = 0.016 and *p* = 0.028, respectively) in cisplatin-treated organoids than in controls (Fig 4D).

NGAL expression was also evaluated in gentamicin- and cisplatin-treated human kidney tubular organoids (S1A Fig). The cisplatin-treated organoids showed significantly higher NGAL expression compared with controls (*p* = 0.004) (S1A Fig). Gentamicin-treated organoids showed higher NGAL expression than controls, but the difference was not statistically significant (*p* = 0.078) (S1A Fig). KIM-1 expression in gentamicin-treated organoids and controls was similar (S1B Fig). In aggregate, these findings indicate that experimental organoid models of normal human kidney cells respond to nephrotoxic substances in a manner similar to that of normal human kidney tubular cells.

## Discussion

The kidney is an architecturally complex organ composed of many cell types and not easily studied *in vivo*. For this reason, simpler research tools are typically used, particularly 2D culture models. Several studies have generated intermediate mesoderm from pluripotent stem cells (i.e., ESCs, induced pluripotent stem cells) that can be used to reconstitute kidney [24, 25]. However, these organoids like nephron progenitors cannot accurately model the complexity of the kidney. While 2D cell-culture models are valuable for the study of renal pathophysiology, 3D-culture models are now considered superior because they can produce a biomimetic environment [2]. Unlike 2D cultures, the more complex tissue elements of 3D cultures have advanced self-organization and enhanced structural capacities [11]. The resemblance of organoids to human kidney imparts greater value as a model. Recently, several researchers have generated kidney organoids (ureteric bud, metanephric mesenchyme, or both) from pluripotent stem cells (embryonic or induced) [11, 26, 27]. The key structural and functional properties of organs replicated through organoid technology may prove useful for exploring human organ pathologies “in a dish” [3].

The value of any cell culture as a disease model or as a target of cell therapy or transplantation depends upon normal genetic/epigenetic sustainability [28]. In terms of safety, the tumorigenic risk of induced pluripotent stem cells limits their clinical use [17], and usage of human ESCs raises ethical concerns. In addition, organoids derived from pluripotent stem cells (vs adult cells) have two distinct drawbacks: 1) time and cost inefficiencies and 2) no assurance of cellular lineage. The adult-cell organoid model we devised circumvents these issues and offers a viable research alternative. To date, organoids have been generated from adult stem cells of mammary glands [29], bone [30], small intestine [31, 32], stomach [13, 33], colon [34, 35], liver [36, 37] pancreas [10, 38], lung [39], prostate [40, 41], salivary gland [42] and tongue [43] but never before from adult kidney cells. Although it is difficult to generate a perfect adult kidney cells derived organoid with a complex structure and individual functions, it is challenging to produce adult kidney organoids while staying from complicated differentiation process that require much time and cost like stem cells.

In the present study, the human kidney tubular organotypic model generated from normal human kidney cells was fully realized and validated. We initially used commercially available tubular cells (primary RPTECs) as controls to gauge the replication of tubular cells in our organotypic model, comparing respective cellular morphologies (Fig 1A). Our organotypic model showed a greater variety of cell types, although most were similar to those generated by primary RPTECs. The morphologies of organoids formed by normal human kidney cells and primary RPTECs in 3D-embedded culture (Fig 1B), were similar. The same result was observed using 3D-on-top cultures (Fig 1B), indicating that the methods are comparable. However, 3D-on-top culture was less demanding than 3D-embedded culture in terms of time, Matrigel use, and ease of imaging and cell purification.

The normal human kidney cells we obtained from patients were used immediately with all of the cells that constitute the kidney for 3D organotypic culture from the onset, whereas primary RPTECs was prepared through the optimal step to acquire pure tubular cells by 2D culture method. Normal human kidney organotypic model more readily showed the formation of tubulocysts than primary RPTECs (Fig 2A and 2B). This finding suggests that our 3D organotypic condition supports more proper kidney cell culture milieu to proliferate and differentiate.

Organoid 3D-structures have been cultivated previously from pluripotent stem cells, neonatal stem cells, and adult stem cells/adult progenitors [44]. To generate human kidney tubular organoids, epidermal growth factor (EGF), Noggin, and Rho kinase inhibitor were used. Thus, our organoid model required only an appropriate environment and was unencumbered by genetic mutations or induction of cellular differentiation. Adult stem cells possess more genomic stability than pluripotent stem cells, and it is genomic stability that confers the greatest advantage in organoid culture techniques [44].

We also measured the amount of Tamm-Horsfall protein expressed by normal human kidney cells in both 2D culture and organotypic culture. Tamm-Horsfall protein is kidney-specific and expressed in renal tubules [21]. Compared with 2D culture, organotypic culture showed greater expression of Tamm-Horsfall protein (Fig 3A), suggesting that human kidney organoids better approximate normal cellular characteristics.

When organoids were dissolved and re-cultured, dome-like structures emerged (Fig 3B and S1 Video), indicating that solute transport and water influx were occurring in these tubular formations [45]. The latter is an important function of renal tubular cells, which underscores the potential to reproduce renal tubular function with human kidney tubular organoids.

Finally, expression levels of KIM-1 and NGAL were assessed after exposing human kidney tubular organoids to gentamicin or cisplatin (Fig 4 and S1 Fig). Gentamicin and cisplatin are commonly used for nephrotoxicity screening. The response shown by human kidney tubular organoids was identical to that documented in the kidneys. Of note, reaction to these particular nephrotoxic drugs is not universally representative. Further research is needed, using a broader range of agents. There were several study limitations. First, it is hard to observe other non-tubular elements (e.g., glomeruli) developed, perhaps because tubules account for the largest proportion of renal mass. On the other hand, conditions used in this study may simply have favored the formation of tubular structures. Variations in culture conditions may be needed to test this hypothesis in future studies.

Another limitation is the drug-induced nephrotoxic injury used for functional validation. The human kidney is a highly vascularized organ, receiving 25% of the cardiac output. Thus, the vascular effects of drugs are an important aspect of nephrotoxicity. Some studies have shown that renal blood flow decreases in kidney damage caused by gentamicin and cisplatin [46, 47]. Therefore, the lack of a vascular component creates a critical void in our model.

Organoid cultures derived from kidney biopsy samples may be of value in studying the pathophysiology of renal epithelium. Clearly, there are potential applications for such models [2]. First, we could use this organotypic technique to determine the mechanisms of renal homeostasis and role of adult stem cells in kidneys. High-throughput screening of related genes and factors would allow us to gain a better understanding of renal physiology. Second, our understanding of the pathophysiology of kidney disease could be broadened. In patients with genetically linked kidney diseases, the use of organoid renal constructs (biopsy generated) or genetic engineering tools, such as Zinc-finger nuclease, transcription activator-like effector nucleases (TALEN), and CRISPR-Cas9 [44], may offer new hope for treatment or a cure. In fact, the cysts that characterize polycystic kidney disease have been observed in kidney organoids derived from human pluripotent stem cells with CRISPR-Cas9-mutated PKD1 or PKD2 genes [48]. A tissue-based organoid model of renal cell carcinoma is also feasible, and would shed light on possible new treatments. Studies of kidney-specific infectious diseases such as polyoma BK virus are also conceivable [49, 50]. Third, a biobank of renal organoids derived from the general population could be used as a source of tissue for nephrotoxicity studies. In some respects, organoids are superior to animal models as drug screening tools because they would eliminating interspecies differences and human heterogeneity. The promise of functional, implantable kidneys represents the pinnacle of organoid potential.

Despite the many imagined uses for organoids, it is important to consider the current limitations. Organoid models are not innervated or vascularized and are devoid of immune cells, so disease processes are only partially represented [3]. Nevertheless, the kidney-specific gene expression, 3D configuration, and response to nephrotoxic drugs they display provide a reasonable platform for studies of renal physiology or drug toxicity screening.

Herein, we have successfully cultured human adult kidney tubular organoids through prototypic cultivation of normal human kidney cells. Our organotypic model replicates kidney-specific features but still needs further development. In this study, we showed that our protocol of organotypic culture was sufficient to promote organoid formation in kidney tubules. Further studies are needed to identify the most suitable conditions for studying kidney physiology, pathophysiology, drug toxicity, and transplantable tissue. This model is a milestone event, moving us closer to realizing precise, patient-oriented clinical treatments.

## Supporting information

S1 Fig Human kidney tubular organoid response to nephrotoxic drugs. (A) Confocal images: immunostaining of neutrophil gelatinase-associated lipocalin (NGAL) in mildly dissolved and re-cultured human kidney tubular organoids after gentamicin or cisplatin treatment, compared with untreated controls (upper panel); quantification of fluorescence intensity of NGAL in gentamicin-treated, cisplatin-treated, or untreated (control) human kidney tubular organoids (lower panel). NGAL (red), anti-NGAL antibody; DAPI (blue), 4′,6-diamidine-2′-phenylindole dihydrochloride. Results expressed as mean (± SD of six independent experiments (n = 6); *p<0.05 vs control. (B) Confocal images: immunostaining of kidney injury molecule-1 (KIM-1) and Tamm-Horsfall protein (THP) in human kidney tubular organoid sections. Gentamicin, gentamicin-treated human kidney tubular organoid; KIM-1 (red), anti-kidney injury molecule-1 antibody; THP (green), anti- Tamm-Horsfall protein (FITC-conjugated); DAPI (blue), 4′,6-diamidine-2′-phenylindole dihydrochloride.
(PDF)

S1 Video Dome-like configuration of human kidney tubular organoid. Confocal 3D-stack of human kidney tubular organoid, as dipicted in Fig 3B. Human kidney tubular organoid expression of Tamm-Horsfall protein (green), pan-keratin (red), and DAPI (blue) nuclear staining.
(AVI)
