# Supplementary figures and images for: Tubular organotypic culture model of human kidney
Source: PLoS One. 2018 Oct 31;13(10):e0206447. doi: 10.1371/journal.pone.0206447 (PMC6209336; doi:10.1371/journal.pone.0206447)

**A****Organoid whole stain**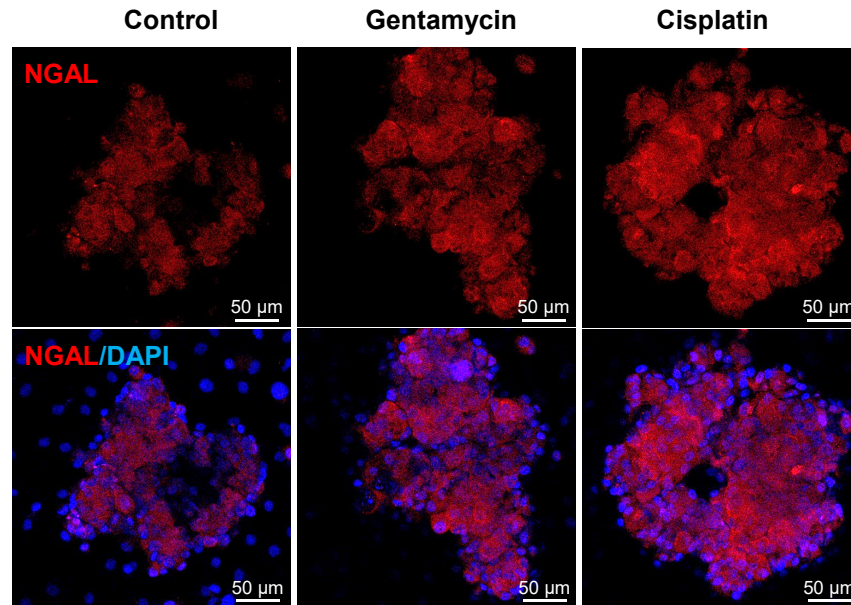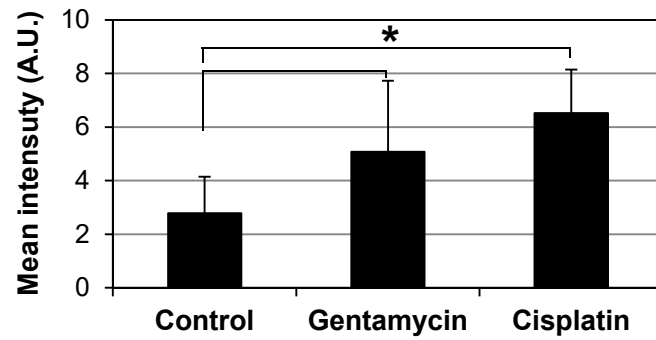**B****Sectioned organoid stain**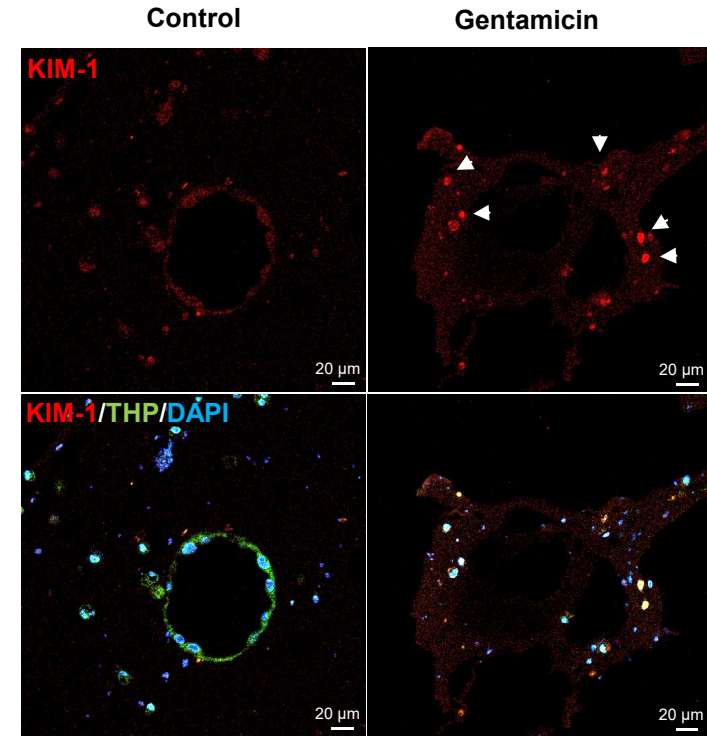

Supplement: S1 Fig — (A) Confocal images: immunostaining of neutrophil gelatinase-associated lipocalin (NGAL) in mildly dissolved and re-cultured human kidney tubular organoids after gentamicin or cisplatin treatment, compared with untreated controls (upper panel); quantification of fluorescence intensity of NGAL in gentamicin-treated, cisplatin-treated, or untreated (control) human kidney tubular organoids (lower panel). NGAL (red), anti-NGAL antibody; DAPI (blue), 4′,6-diamidine-2′-phenylindole dihydrochloride. Results expressed as mean (± SD of six independent experiments (n = 6); *p<0.05 vs control. (B) Confocal images: immunostaining of kidney injury molecule-1 (KIM-1) and Tamm-Horsfall protein (THP) in human kidney tubular organoid sections. Gentamicin, gentamicin-treated human kidney tubular organoid; KIM-1 (red), anti-kidney injury molecule-1 antibody; THP (green), anti- Tamm-Horsfall protein (FITC-conjugated); DAPI (blue), 4′,6-diamidine-2′-phenylindole dihydrochloride. (PDF) [file pone.0206447.s001.pdf]
